# High expression of H2A histone family member Y promotes the proliferation and autophagy of hepatocellular carcinoma cells

**DOI:** 10.1080/21655979.2022.2065761

**Published:** 2022-04-26

**Authors:** Jiasheng Lei, Shuoshuo Ma, Wanliang Sun, Dongdong Wang, Zheng Lu, Dengyong Zhang

**Affiliations:** Department of Hepatobiliary Surgery, The First Affiliated Hospital of BengBu Medical College, BengBu, Anhui, China

**Keywords:** Hepatocellular carcinoma, prognosis, proliferation, autophagy, bioinformatics

## Abstract

Hepatocellular carcinoma is a common malignant tumor and the third most common cause of cancer-related deaths. In this study, we selected H2AFY as a potential oncogene from three online databases, and verified differential expression between normal and liver cancer tissues. Moreover, H2AFY expression was significantly correlated with the clinical characteristics and the survival of liver cancer patients. H2AFY expression was correlated with poor prognosis of liver cancer patients. H2AFY expression was also significantly higher in liver cancer cells. Knockdown and overexpression of H2AFY in liver cancer cells showed that H2AFY promoted the proliferation and clone formation of liver cancer cells but had no significant effects on the migration and invasion ability of liver cancer cells. Western blot analysis, immunohistochemistry, and immunofluorescence double staining confirmed that H2AFY upregulated LC3 and p62 expression in liver cancer tissues and cells. In conclusion, H2AFY is highly expressed in liver cancer cells and tissues, and promotes the proliferation and autophagy of liver cancer cells. H2AFY is a potential target for liver cancer therapy.

**Abbreviations**: APLF: aprataxin pnk-like factor; HCC: Hepatocellular carcinoma; H2AFY: H2A histone family member Y

## Graphical abstract

**Figure:**
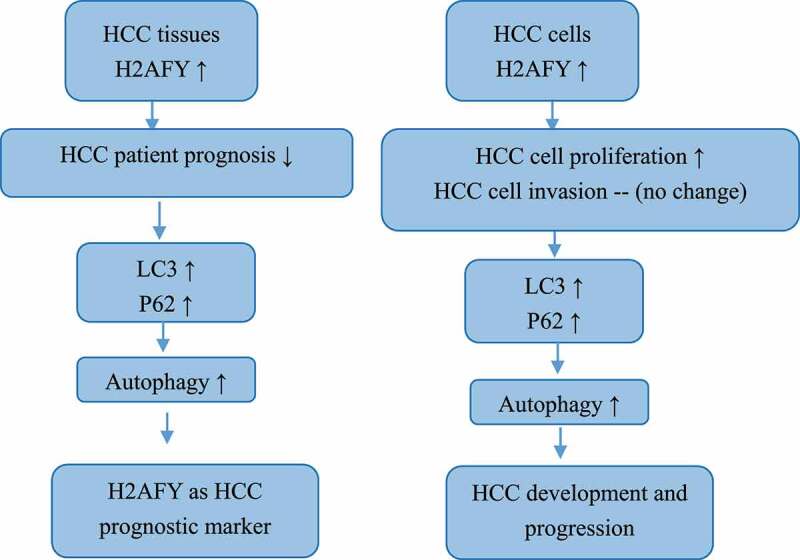

## Highlights

- H2AFY is highly expressed in liver cancer cells and tissues
- H2AFY was associated with poor prognosis of liver cancer patients
- H2AFY promoted the proliferation and autophagy of liver cancer cells.

## Introduction

Hepatocellular carcinoma (HCC) is a common primary malignant tumor that represents the vast majority of liver cancers with a high mortality rate worldwide [1]. Because liver cancer progresses rapidly and tumor-related mortality is high, liver cancer is recognized as a major global health problem [2]. The prognosis with early diagnosis of HCC is much better than that with later diagnosis. In the early stage of HCC, surgical resection and transplantation can significantly increase the survival rate of patients [3].

H2A histone family member Y (H2AFY) is a mutant histone encoded by H2AFY gene. H2AFY forms a more stable chromosome structure in the process of chromatin structure regulation, which leads to the X chromosome silencing function [4]. Histones and their metabolism are regulated by histone chaperones, which are important regulators of epigenetic regulation [5]. For example, downregulation of the histone chaperone aprataxin pnk-like factor (APLF) increases E-cadherin expression. E-cadherin is known to inhibit cancer metastasis. Attached colonization and downregulation of APLF expression accelerates the loss of H2AFY in E-cadherin promoter region. This regulatory mechanism may have important implication for epigenetic regulation and tumor metastasis [6]. Therefore, understanding the role of H2AFY in HCC may help develop novel therapeutic strategies.

Autophagy is an evolutionary conserved intracellular mechanism that helps eukaryotic cells maintain homeostasis [7]. Autophagy plays a vital role in tumorigenesis, with dual function in inhibiting and promoting cell survival [8]. Autophagy mainly relies on two ubiquitin-like systems: the binding process of ATG12 and the modification process of LC3. Under the action of E1 and E2 enzymes, ATG12 forms a multibody complex of ATG12-ATG5-ATG16 [9]. In liver cancer, the autophagy process may have different functions at different stages of cancer development. Owing to the self-immune system and cell self-monitoring function, it is easier to activate the autophagy of liver cancer cells in the early stage of liver cancer; this increases the unprogrammed death and elimination of liver cancer cells. In this case, autophagy generally plays an important role in maintaining the homeostasis of the body and preventing disease. However, in the middle and late stages of liver cancer, the body is unable to eliminate liver cancer cells, and the rapid proliferation of these cells becomes inevitable. In this case, autophagy becomes a cancer-promoting factor to provide energy and raw materials for liver cancer cells. Therefore, in-depth understanding of the complex mechanism of liver cancer autophagy would be beneficial to the treatment and prevention of liver cancer.

In this study, we aimed to detect the expression of H2AFY in HCC and examine the association of H2AFY expression with the prognosis of HCC patients. In addition, we aimed to investigate the function of H2AFY in the regulation of autophagy in HCC.

## Materials and methods

### Data collection

The mRNA expression profiles and clinical data of HCC were downloaded from The Cancer Genome Atlas (TCGA; https://portal.gdc.cancer.gov). In addition, the mutation profiles of patients were obtained from TCGA. GSE14520, GSE76427, and GSE8648 datasets were downloaded from the Gene Expression Omnibus (https://www.ncbi.nlm.nih.gov/geo). The cancer tissues and adjacent tissues were sampled from patients with liver cancers at the First Affiliated Hospital of Bengbu Medical College. This study was approved by Ethics Committee of Bengbu Medical College (Approval No. 2017059, Supplementary file), and all patients provided written informed consent.

### Bioinformatics analyses

Oncomine and GEPIA databases were used to select target gene H2AFY, and GEPIA and Kaplan-Meier Plotter databases were used to analyze the correlation between H2AFY and poor prognosis of liver cancer. From TCGA database, the mRNA expression profiles of 424 liver cancer samples (50 normal samples, 374 tumor samples) and the clinical data of 337 tumor samples (3 samples without clinical data, 6 cases without survival data, 5 cases without tumor pathological staging, and 23 cases of clinical staging data defects were removed) were downloaded. The gene expression matrix of GSE14520, GSE76427, and GSE8648 datasets and TCGA dataset were analyzed using GraphPad Prism 8 to verify the differences in H2AFY expression in normal tissues and tumor tissues. GEPIA database and GraphPad Prism 8 were used to verify the correlation between H2AFY expression and clinical characteristics. According to Spearman’s correlation analysis, the top 300 differentially expressed genes that were significantly related to H2AFY were selected, and the GSEA algorithm was used to predict the cellular functions and signaling pathways. The top 20 significantly enriched signals were selected.

### Cell culture

L02, HepG2, and SMMC-7721 human liver cancer cell lines were purchased from the Cell Bank of Shanghai Institute for Biological Sciences. L02 and HepG2 cells were cultured in Dulbecco’s modified Eagle medium containing 10% fetal bovine serum (FBS) and 1% penicillin-streptomycin mixture, and SMMC-7721 cells were cultured in RPMI-1640 containing 10% FBS and 1% penicillin-streptomycin mixture. All cell lines were maintained in a humidified incubator at 37°C with 5% CO_2_, and they were passaged using 0.25% trypsin at a confluence of 80–90%. Cells were transfected with siRNAs for H2AFY using lipofectamine 2000 reagent as described previously [10].

### qRT-PCR

Total RNA was extracted from cells or tissues using TRIzol® reagent according to the manufacturer’s instructions. cDNA was synthesized using reverse transcription kit, and qPCR was conducted using a StepOnePlus Real-time PCR instrument with GAPDH as the internal reference. The reaction procedure was as follows: predenaturation at 95°C for 30 min, denaturation at 95°C for 5 s, and annealing at 60°C for 10–15 s (40 cycles). The relative expression levels of the target gene were calculated using the 2^−ΔΔCq^ method. Each sample was run in triplicate, and all experiments were repeated independently three times. The primer sequences used were as follows: H2AFY forward, 5’-TTCGGGGTGAGGAGGATTAACT-3’; H2AFY reverse, 5’-GTACTTGGGGTGGCCTTTCTT-3’; GAPDH forward, 5’-GGAAGGAAATGAATGGGCAGC-3’; and GAPDH reverse, 5’-CAGGGTTAGTCACCGGCAG-3’.

### Western blot analysis

Total proteins were extracted from cells or tissues using cell lysis buffer (Beyotime Institute of Biotechnology), and the protein concentration was determined using the bicinchoninic acid assay method. The protein samples (30 µg per lane) were separated using 10%–12% SDS-PAGE, transferred onto membranes, and then blocked with 5% skim milk at room temperature for 2 h. The membranes were incubated with primary antibodies (all from Abcam, Cambridge, UK) overnight at 4°C. The membranes were then incubated with secondary antibody at room temperature for 2 h. After washing with Tris buffer saline/0.1% Tween-20, the bands were visualized using enhanced chemiluminescence solution, and images were captured using a gel imager. Gray values were analyzed using Bio-Rad Image Lab Software 5.1.

### Cell plate cloning

About 1,000 HepG2 cells and SMMC-7721 cells were planted in six-well plates, and 2 ml of cell culture medium was added to each well. At about the 14th day, a clonal cell population became visible to the naked eye. The clonal culture was washed and fixed with 4% paraformaldehyde. The clones were stained with GIMSA for 15–30 min, and the clone formation rate was calculated as follows: clone formation rate = number of clones/number of clones inoculated × 100.

### CCK-8 assay

Five 96-well plates were marked as days 0, 1, 2, 3, and 4 according to the test days. Cells were seeded in 96-well plates at the density of 9,000 cells/well. On day 1–4, 10 μl of CCK-8 reagent was added to each well, and the cells were incubated for 2 h in an incubator. Subsequently, the absorbance value in each well was measured at 450 nm with a microplate reader. Finally, a growth curve was drawn based on OD values.

### Scratch test

Cell migration was evaluated using scratch test as described previously [11]. Briefly, cells were seeded in 6-well plates and cultured for 2–3 days in an incubator to a confluence of 90%. A scratch was made in the middle of each well using a 200 μl pipette tip. The width of the scratch was observed and imaged. After culture for 24–36 h, the width of the scratch was again observed and imaged, and the differences in scratch width were compared among the groups.

### Transwell assay

Cell invasion was evaluated using transwell assay as described previously [12]. Briefly, cells were seeded in. Transwell precoated with Matrigel (Corning, USA) was added into 24-well plates. The cells were seeded to the upper chamber, and 800 μl of medium containing 10% FBS was loaded to the lower layer. The cells in the upper chamber were carefully wiped with cotton swab, fixed with 4% paraformaldehyde for 20 min, washed and stained with 0.1% crystal violet for 20 min. The numbers of cells in five random fields were counted under a microscope.

### Immunofluorescence staining

Tissue slices were blocked with 200 μl of blocking solution for 2 h. The slices were then incubated with primary antibodies for H2AFY, LC3, and p62 overnight at 4°C. The slices were washed and then incubated with fluorescein isothiocyanate and Rho labeled secondary antibodies for 2 h. Finally, the slices were washed, dried, dripped with glycerol (including Hoechst nuclear stain), mounted on slides, and images were taken at 4°C in the dark.

### Statistical analysis

The data were expressed as the mean ± standard deviation. Statistical analysis methods included t-tests and one-way ANOVA, which were used to compare the differences. P values <0.05 were considered statistically significant.

## Results

### Overexpression of H2AFY in liver cancer

**Figure 1.**
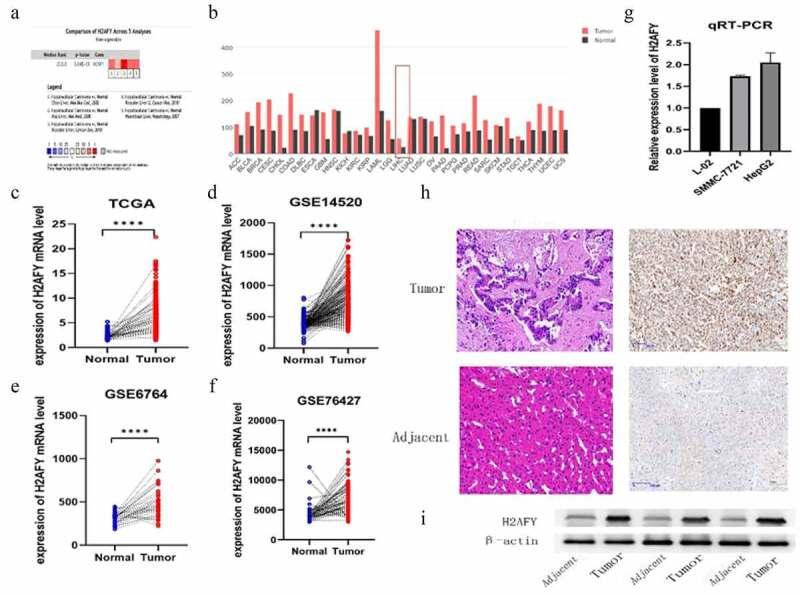
The expression of H2AFY in liver cancer tissues. a-b. H2AFY was highly expressed in liver cancer tissues in the ONCOMINE and GEPIA databases. c-f. The expression levels of H2AFY in four different gene sets. g. The expression level of H2AFY in liver cancer cell lines was higher than that in normal liver cell lines. h. Histochemical analysis of H2AFY in liver cancer tissues. Left: Hematoxylin and eosin staining. Right: Immunohistochemical staining of H2AFY. i. Western blot analysis of H2AFY in liver cancer tissues.

**Figure 2.**
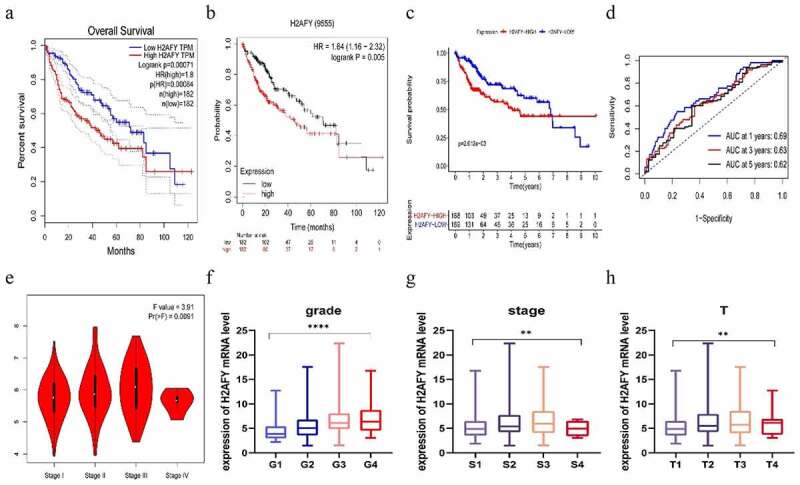
H2AFY was associated with poor prognosis of liver cancer. a-d. The poor survival time associated with H2AFY in the prognosis of liver cancer patients (P = 0.00071, P = 0.005, and P = 2.612e−03) as well as the 1-, 3-, and 5-year survival rates (P = 0.69, P = 0.63, and P = 0.62). e-h. The expression level of H2AFY was associated with the clinical stage and grade of liver cancer patients.

According to the Oncomine database, H2AFY was overexpressed in liver cancer tissues (Figure 1(a)). Based on GEPAI database, H2AFY expression levels in liver cancer tissues were significantly higher than those in normal tissues (Figure 2(b-g)). We downloaded the transcriptome expression chips of liver cancer patients from TCGA and the GEO database to verify TCGA (Figure 1(c)), GSE14520 (Figure 1(d)), GSE6764 (Figure 1(e)), and GSE76427 (Figure 1(f)). In these gene sets, H2AFY was overexpressed in liver cancer tissues. To detect expression level of H2AFY, we collected three clinical liver cancer samples from patients after resection, including cancerous tissues and adjacent tissues, and performed immunohistochemical staining (Figure 1(h)). H2AFY protein levels in liver cancer tissues were significantly higher than those in adjacent tissues (Figure 1(i)).

### H2AFY was associated with poor prognosis of liver cancer

To explore the role of H2AFY in the prognostic development of liver cancer, we used the GEPIA and Kaplan-Meier Plotter databases; H2AFY was found to be a poor prognostic marker in liver cancer development (Figure 2(a,b)). We performed survival analysis for H2AFY (Figure 2(c,d)) and correlations of clinical characteristics from 374 liver cancer samples from TCGA. The expression level of H2AFY was associated with clinical and pathological stages and survival time (Figure 2(e,h)).

### Knockdown of H2AFY inhibited the proliferation of liver cancer cells

**Figure 3.**
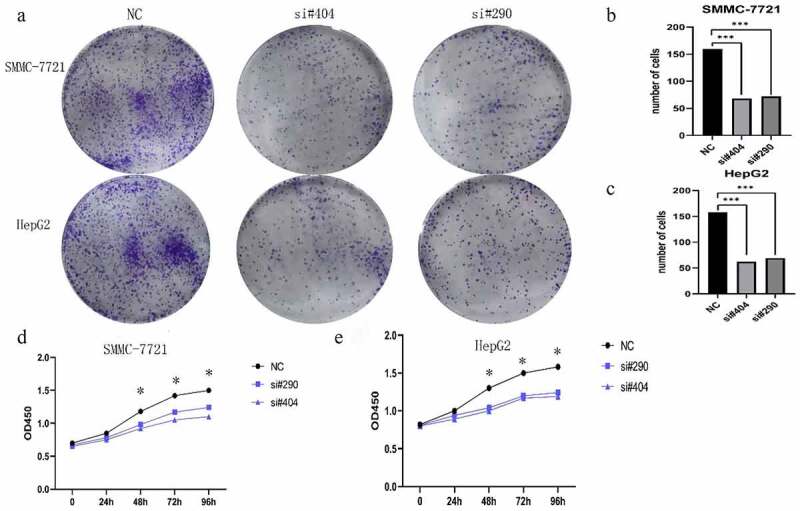
H2AFY knockdown inhibited the proliferation of liver cancer cells. a. Cell cloning assay of SMMC-7721 and HepG2 cells with the knockdown of H2AFY. b-c. The number of cell clones in each well. d-e. CCK-8 assay of SMMC-7721 and HepG2 cells with the knockdown of H2AFY.

To determine the role of H2AFY in the proliferation of liver cancer cells, we conducted cell plate cloning and CCK-8 assays using two liver cancer cell lines: SMMC-7721 and HepG2. Two sets of siRNAs were used to knockdown H2AFY in liver cancer cells. The number of cell colonies significantly reduced after knockdown of H2AFY (Figure 3(a-c)). Moreover, the proliferation ability of liver cancer cells decreased after knockdown of H2AFY (Figure 3(d-f)).

### H2AFY knockdown did not affect migration and invasion of liver cancer cells

**Figure 4.**
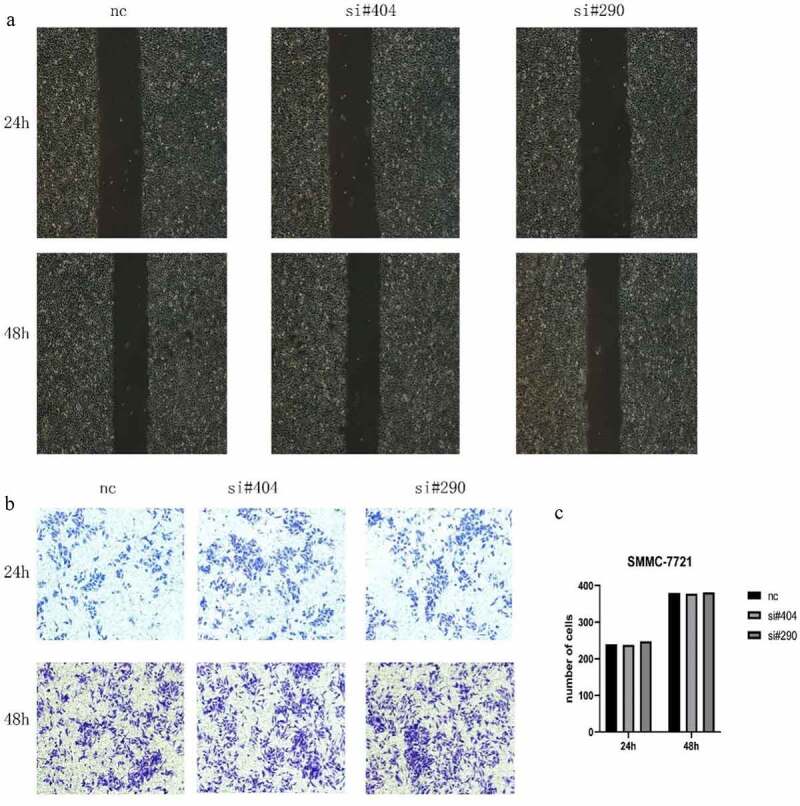
H2AFY knockdown did not affect the metastasis ability of liver cancer cells. A. Scratch assay of SMMC-7721 cells treated with two sets of H2AFY siRNAs. B-C. Transwell assay of SMMC-7721 cells treated with two sets of H2AFY siRNAs.

To determine the role of H2AFY in liver cancer invasion, we used SMMC-7721 cells for scratch and Transwell tests. As shown in Figure 4(a), there was no significant difference in the change in scratch width after H2AFY knockdown. Transwell test showed that the number of invaded liver cancer cells was not significantly different among the groups (Figure 4(b,c)).

### H2AFY regulated the autophagy of liver cancer cells

**Figure 5.**
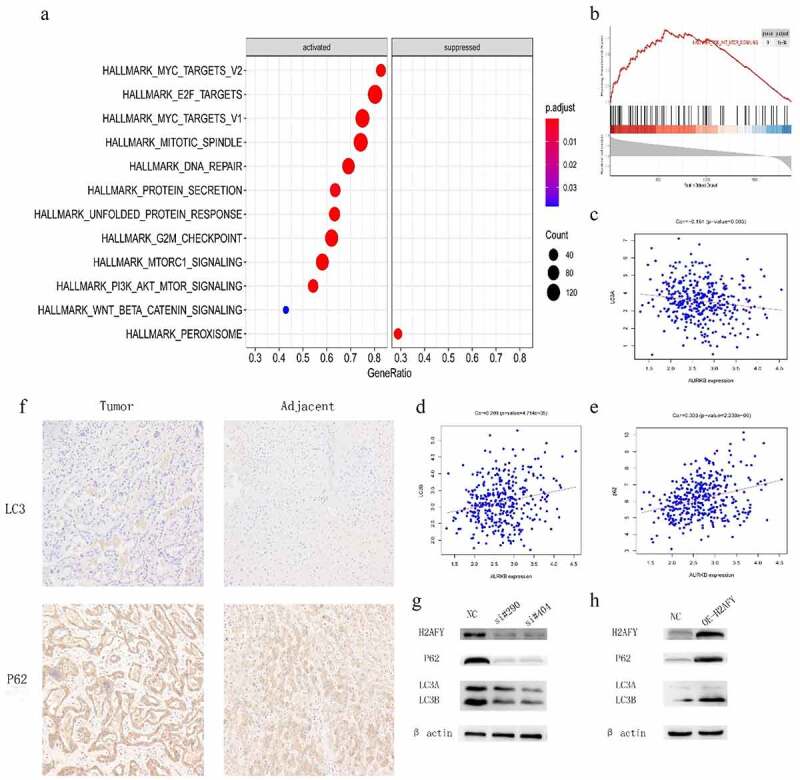
H2AFY regulated the autophagy of liver cancer cells A-B. The enriching of H2AFY-related genes according to Spearman’s correlation coefficient. C-E. The correlation between LC3 and P62 proteins and H2AFY protein. F. The expression levels of LC3 and P62 in liver cancer tissues. G. LC3 and P62 protein levels after knockdown of H2AFY.H. LC3 and P62 protein levels after the overexpression of H2AFY.

To further explore the role of H2AFY in liver cancer, we used Rstudio to analyze the genome-wide transcription of 374 liver cancer samples, and used the GSEA algorithm to determine the functions and pathways (Figure 5(a)). We found that H2AFY was positively correlated with PI3K-AKT-MTOR pathway (Figure 5(b)). Furthermore, H2AFY significantly affected the expression of the autophagy markers LC3 and p62 (Figure 5(c-e)). Therefore, we overexpressed H2AFY in SMMC-7721 cells and verified that the expression of LC3 and p62 significantly changed with the change of H2AFY expression (Figure 5(g,h)).

**Figure 6.**
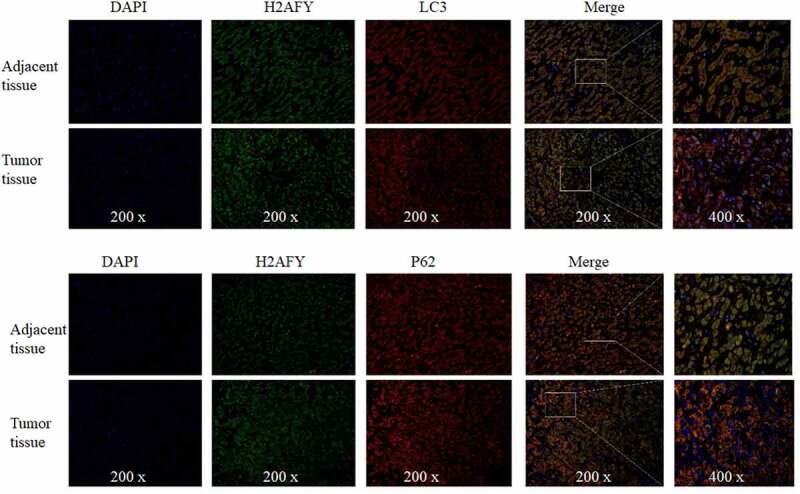
The colocalization of LC3 and P62 with H2AFY in liver cancer tissues. DAPI indicated nuclear staining (blue).

Immunohistochemical staining showed that both LC3 and p62 were increased in liver cancer tissues (Figure 5(f)). Finally, we performed immunofluorescence double-staining experiments on the cancer tissues and adjacent tissues of three groups of patients. H2AFY was colocalized with LC3 and p62 in the cytoplasm (Figure 6).

## Discussion

HCC is one of the most common malignant tumors in the world and accounts for >80% of liver cancer cases. HCC is highly malignant, recurrent, drug resistant, and often diagnosed at an advanced stage. Therefore, there is an urgent need to better understand molecular mechanisms of HCC. In this study, based on bioinformatics analysis, we found that H2AFY was highly expressed in HCC tissues and H2AFY expression was associated with poor prognosis of HCC patients. In addition, using HCC cell lines as experimental model we showed that H2AFY was highly expressed in HCC cells and promoted the proliferation and autophagy of HCC cells. We verified the difference in the expression of H2AFY in normal tissues and liver cancer tissues using four expression datasets from TCGA and the GEO database. We also used the GEPIA and Kaplan-Meier Plotter databases and TCGA data to analyze the correlation of H2AFY and the prognosis of liver cancer patients. As the expression levels of H2AFY increased, the clinical and pathological stages of patients increased and the prognostic survival time shortened, implying that H2AFY is associated with poor prognosis of liver cancer patients.

To confirm H2AFY as an oncogene in HCC, we used three cell lines: L02, SMMC-7721, and HepG2. qPCR and Western blot analysis showed that the mRNA and protein levels of H2AFY in SMMC-7721 and HepG2 cell lines were higher than those in L02 cell lines. In addition, immunohistochemical staining showed that H2AFY protein levels were high in liver cancer tissues. Despite high expression level of H2AFY in liver cancer tissues, it is unclear how the increase in H2AFY expression would affect tumor phenotype (e.g., the proliferation, invasion, and migration abilities) of liver cancer cells.

Therefore, we interfered with H2AFY expression in HCC cell line SMMC-7721. CCK-8 assay and cell clone assay showed that after interfering with H2AFY, the proliferation and growth of liver cancer cells decreased significantly. These data suggest that H2AFY plays a specific role in promoting the proliferation and growth of liver cancer cells. However, scratch test and Transwell assay showed that H2AFY had no significant effects on the invasion and migration ability of liver cancer cells. We postulate that H2AFY may not participate in the invasion and migration of liver cancer cells and has little effect on the distant metastasis of liver tumor.

To determine the cellular functions in which H2AFY participates, we used bioinformatics technology to predict the GO and KEGG terms related to H2AFY. H2AFY was positively correlated with PI3K-AKT-mTOR activation. The main molecules of autophagy are LC3 and p62 [13]. LC3 plays an important role in helping cancer cells against the external environment [14]. The p62 protein molecule is a multifunctional signal transduction center and an autophagy aptamer with multiple binding molecules. It can stimulate mtorc1-dependent nutrient sensing, NF-κb-mediated inflammation, and NRF2-activated antioxidant defense. The signal process and combination with LC3 accelerate the autophagy degradation process of ubiquitination products [15]. p62 is accumulated in many liver diseases, including nonalcoholic steatohepatitis and HCC [16,17]. Although lipopolysaccharide-induced p62 accumulation can promote the clearance of autophagy-like aggregates in hepatocytes, autophagy damage leads to p62 accumulation, which further damages autophagy through mTORC1 activation. Notably, p62 is found in hepatocytes, and accumulation can promote the development and recurrence of liver cancer [18,19]. Therefore, we analyzed the correlation between H2AFY and the expression of LC3 and P62 in liver cancer tissues, and found a positive correlation with both LC3 and P62. Therefore, we conclude that H2AFY is involved in the regulation of the expression of LC3 and P62.

Autophagy presents different functional states in different stages of HCC development. Usually, in the early stage of HCC, autophagy inhibits tumor growth by enhancing the degradation of defective proteins and inhibiting the accumulation of genotoxic-free radicals [20]. In contrast, in the advanced stage of HCC, the cells are usually in a state of ischemia and hypoxia, and autophagy helps with low nutrition. Hypoxic liver cancer cells enhance protein degradation to obtain nutrient and increase the ability to resist the external environment. This function allows liver cancer cells to survive and continue to proliferate in harsh environments [21]. In this study, LC3 and p62 proteins were detected in the liver cancer cell line SMMC-7721, and the expression of H2AFY was positively correlated with the levels of LC3 and p62 proteins. Furthermore, immunohistochemical testing revealed that LC3 and p62 protein levels in liver cancer tissues were significantly higher than those in normal tissues. Immunofluorescence double staining showed that H2AFY was colocalized with LC3 and p62 proteins in the cytoplasm of liver cancer cells. Although H2AFY mainly functions in the nucleus and silences gene expression, we demonstrated that H2AFY was present in the cytoplasm to bind LC3 and p62 proteins. The increase in H2AFY protein levels in liver cancer tissues may enhance the autophagy function of liver cancer cells.

We acknowledge that our study has some limitations. First, our study is based on bioinformatics analysis, and we did not collect data or follow-up patients after liver cancer surgery. Therefore, the correlation between H2AFY and the clinical characteristics/poor prognosis of liver cancer patients must be verified. Second, we did not determine how H2AFY regulates the expression of LC3 and p62 proteins, and the mechanism that H2AFY promotes autophagy in liver cancer.

## Conclusion

H2AFY is highly expressed in liver cancer cells and tissues, and promotes the proliferation and autophagy of liver cancer cells. H2AFY is a potential target for liver cancer therapy.
